# Trends in Overall and Menthol Market Shares of Leading Cigarette Brands in the USA: 2014–2019

**DOI:** 10.3390/ijerph19042270

**Published:** 2022-02-17

**Authors:** Erin J. Miller Lo, William J. Young, Ollie Ganz, Eugene M. Talbot, Richard J. O’Connor, Cristine D. Delnevo

**Affiliations:** 1 Rutgers Center for Tobacco Studies, New Brunswick, NJ 08901, USA; william.j.young@rutgers.edu (W.J.Y.); og96@cts.rutgers.edu (O.G.); et304@cts.rutgers.edu (E.M.T.); 2 Roswell Park Comprehensive Cancer Center, Buffalo, NY 14203, USA; richard.oconnor@roswellpark.org

**Keywords:** cigarettes, smoking, tobacco use trends, menthol, combustible tobacco, tobacco marketing, cigarette brands

## Abstract

Many factors can shift cigarette brand preference, and surveillance is an important tactic to inform regulatory strategy. The objective of this study was to identify shifts in top brands’ overall and menthol market share from 2014 to 2019. We used data from the National Survey on Drug Use and Health public use datasets, which are a nationally representative, cross-sectional survey of people aged 12+ in the USA. In our analysis of top brands, we accounted for consumption patterns and computed the percent change in market share for each brand. We observed that overall market share declined for nearly all brands, though top moderately priced brands gained share. Half of the top brands with menthol styles grew in menthol market share. We observed three primary shifts in the cigarette market: brands that gained the most menthol market share were brands with both menthol and non-menthol in their product lineups; menthol contributed substantially to discount brands’ market share increases; the two premium brands that employed “natural” descriptors experienced increased market share. Research should continue to focus on trends that influence cigarette market share, as the cigarette market in the USA is likely to look very different in five years than it does today.

## 1. Introduction

For nearly a quarter of a century, most traditional advertising channels have been closed to cigarette manufacturers by the Master Settlement Agreement of 1998 (MSA) [1]. Despite these barriers, the tobacco industry continues to be invested in promoting products to consumers through any permissible channel, and cigarettes remain one of the most heavily promoted products in the United States, with industry-wide advertising expenditures of more than USD 7.5 billion in 2019 [2]. 

In the years since the MSA and the subsequent Tobacco Control Act (TCA) of 2009 [3,4], which introduced additional restrictions to advertising and promotion, and the Children’s Health Insurance Program Reauthorization Act of 2009 (S-CHIP), which introduced a 61.6 cent federal excise tax (FET) increase on cigarettes [5], most tobacco promotional spending has been spent on price discounts. Over time, as marketing efforts have largely moved to the point of sale [2], price promotion has been achieved via money paid directly to retailers to offset the consumer price, and to wholesalers for promotional services [6]. In 2019, this type of marketing accounted for the vast majority of the industry’s advertising and promotional expenditures, totaling USD 7.125 billion, or 93.4% of spending. 

From 2000 to 2011, menthol cigarettes’ market share was relatively stable, compared with non-menthol cigarettes, which declined over that period [7,8,9]. Moreover, a 2020 study of two decades of cigarette consumption and sales data demonstrated that menthol cigarettes are becoming increasingly dominant in the tobacco marketplace: menthol cigarette market shares have increased, while non-menthol cigarette shares have decreased, and 91% of the total decrease in cigarette consumption in the US from 2009 to 2018 can be attributed to non-menthol cigarettes [10].

The inclusion of menthol in a separate analysis is important to identify any shifts in preference from non-menthol to menthol brands, as menthol cigarettes pose a particular public health risk even beyond those of non-menthol cigarettes. Data from decades of research indicate that menthol in cigarettes may be associated with increased smoking initiation and may promote greater addiction among those who regularly use menthol cigarettes. Additionally, menthol cigarette smokers may find cessation especially difficult, as many appear to be even more nicotine dependent than their non-menthol cigarette-using counterparts [11].

Marketing practices, regulation, and taxation are among many factors identified as having an influence on the popularity of brands in the cigarette marketplace [3], and identification of shifts in brand preferences can inform regulatory strategy and provide insight into the tobacco industry’s promotional tactics. To that end, the aim of this analysis was to update a previous study [3] of trends in market share for leading cigarette brands in the United States from 2002 to 2013 and examine the subsequent six years of trends from 2014 to 2019. The focus of the present analysis is on the top cigarette brands overall, as well as menthol brands.

## 2. Methods

### 2.1. Data Source

The present study utilized data from the National Survey on Drug Use and Health (NSDUH) public-use datasets. A nationally representative, cross-sectional survey, NSDUH estimates substance use behaviors in the civilian, non-institutionalized population aged 12 and older by surveying approximately 70,000 respondents in the United States annually. Data from 2014 to 2019 were used for this study and were analyzed in 2021 [12].

To arrive at nationally representative estimates of substance use and mental illness, NSDUH employs an independent, multistage area probability sample for each state and the District of Columbia. Basic questions such as demographics are administered using computer-assisted personal interviewing while sensitive items, such as those pertaining to substance use, are administered via audio computer-assisted self-interviewing [12]. Full information on the methodology of the most recent iteration of NSDUH is available online at https://www.samhsa.gov/data/report/2019-methodological-summary-and-definitions (accessed on 28 June 2021).

### 2.2. Cigarette Brand Measures

The following analyses are based on 62,772 current smokers, aged 12 and older, that reported having smoked at least part of a cigarette in the 30 days prior to completing the survey. No distinctions were made with regard to single or poly-tobacco use. These individuals were asked to report the cigarette brand that they use most often. For each year between 2014 and 2019, we examined the frequency distribution of this variable and restricted our focus to the top 10 cigarette brands each year, resulting in a shortlist of 15 brands across the entire time period. This analysis was repeated for the subgroup of current smokers who reported smoking menthol cigarettes. Specifically, the item (CIG30MEN) asked past 30-day smokers, “Were the cigarettes you smoked during the past 30 days menthol?” Focusing on the top 10 brands for each year among this subpopulation resulted in a shortlist of 14 brands. Importantly, this paper only examines brand families (e.g., Camels), not subbrands (e.g., Camel Reds), as NSDUH does not collect data on precise brand variants.

### 2.3. Weight Adjustment

NSDUH’s default survey weight (ANALWT_C) weights the sample to be representative of the US population. As our analysis focused on cigarette brand market share, however, we adjusted the weighting variable by multiplying it by the average number of cigarettes consumed each day and the total number of days that participants reported smoking in the past month. Accordingly, the adjusted weight reflects the number of cigarettes consumed by current smokers in the past 30 days. It, therefore, accounts for consumption patterns among these individuals and produces a better picture of the market in terms of the actual number of cigarettes smoked. This weighting technique has been used in the past for analyses of brand preferences using NSDUH data [3].

### 2.4. Statistical Analysis

Analyses were conducted in SPSS version 27. Applying the new weighting variable, we ran frequency distributions of the “usual brand” variable for each year individually, overall, and for menthol. We also examined changes in market share between 2014 and 2019 by computing the percent change in market share for each brand over this six-year period.

## 3. Results

During the time period from 2014 to 2019, 15 brands comprised 75% of the cigarette market (see Table 1).

Overall, market share declined for nearly all brands, including perennial market share leader Marlboro, but grew for several brands including American Spirit, Winston, Basic, Newport, and L&M. Despite a small loss in market share, Marlboro’s share of the overall cigarette market remained the highest among brands, at an average of 39% each year. Pall Mall, which had a decade of tremendous growth from 2002 to 2013 [3], stabilized at about 8% market share each year. Camel’s market share declined 13% from 2014 to 2019 and took fourth place in the overall market.

Amid these losses, some brands experienced growth in market share. Newport, known largely for menthol, continued to hold the second-highest overall cigarette market share among brands during this time, gaining 17.6% over 2014. Moreover, two premium brands, Natural American Spirit and Winston, saw their market shares increase 97% and 16%, respectively. Lastly, while some deep-discount cigarettes appear to be losing share, other moderately priced brands gained share during the six-year period. Three Altria brands increased in market share: value brand L&M increased 27.1%, and Eagle, marketed as “America’s #1 Discount Brand,” grew 1407.1% from 2014 to 2019, surpassing the company’s more established discount brand Basic. Basic also grew a substantial 81% during the same timeframe.

Half of the top brands with menthol styles grew in menthol market share, while the other half declined (Table 2). Discount brand Basic had the greatest growth, at 168%, its menthol market share growth outpacing its overall market share growth. Natural American Spirit similarly gained substantial menthol market share during this time period, 88%. Top, a roll-your-own-brand, gained 69% in menthol market share, despite a loss in overall market share. Value brand L&M gained 49% in menthol market share.

Top brands in the menthol category continue to be premium brands Newport and Marlboro, and displacing Camel in third place was Pall Mall. Newport is one of the best-known brands of menthol cigarettes and maintained its top spot, with 6% growth among menthol styles. Marlboro gained 18.1% from 2014 to 2019 and remains second in market share among menthol styles. Pall Mall, which has been a strong player in the menthol category, displaced Camel for third place in menthol market share in 2019, with a growth of 4.3%. Brands that lost large amounts of menthol market share from 2014 to 2019 include menthol-only brands Salem and Kool (−60% and −18%, respectively), women-oriented brands (e.g., brands developed specifically for women) Virginia Slims and Misty (−58% and −40%, respectively), as well as discount brands USA Gold and Maverick (−38% and −18%, respectively).

Notably, Salem and Kool, once top brands in the menthol market, had been in decline for at least a decade before 2014 [3] and were acquired in 2015 by Imperial Brands.

In general, youth and young adults, who comprised a small percentage of smokers in 2014, comprised an even smaller proportion in 2019 (Table 3). Subsequently, across nearly all brands, there were shifts in demographic profiles with respect to age, such that the average ages of smokers were older for these brands in 2019 than they were in 2014. The one exception to this is Winston, which saw an increase in share among youth and young adults. Certain brands are “younger brands” such that the majority of smokers of Marlboro, Newport, Camel, and L&M are under the age of 50, whereas other top brands (Pall Mall, Winston, Eagle, Basic, and Kool) have the majority of their smokers at age 50 or older. Interestingly, while Natural American Spirit skews younger overall, their smokers have become older over time: in 2014, 20.2% of Natural American Spirit’s smokers comprised those aged 50 years old or older, but by 2019, that proportion had risen to 32.6%.

Of all the top 10 brands, Natural American Spirit had the greatest proportion of male smokers—about 7 out of 10 in 2014, and 6 out of 10 in 2019.

With respect to race and ethnicity, the overwhelming majority of people that smoked identified as White and Non-Hispanic in both 2014 and 2019. Two notable exceptions, Newport and Kool, historically predominately menthol brands, had a higher proportion of individuals that identified as Black, Non-Hispanic. Compared with 2014, the percentage of those who smoked Newport and identified as White increased slightly in 2019, while the percentage of Black Newport smokers decreased. Lastly, over time, those that smoked Natural American Spirit were less likely to be Hispanic in 2019 than in 2014.

With respect to income, premium brands such as Marlboro, Camel, and Natural American Spirit had a greater proportion of individuals who earned USD 75,000 or more annually; in contrast, among the discount brands such as Eagle and Basic, more individuals were likely to earn less than USD 30,000 annually. Notably, one brand, L&M, seems to have expanded the income diversity of their smokers, reaching more of those with higher incomes in 2019, compared with 2014.

Finally, daily smoking was more common among those who smoke Pall Mall, L&M, Winston, Eagle, and Basic, where at least two out three that smoked the brands in 2019 reported smoking daily. In sharp contrast, the majority of those that report Natural American Spirit as their usual brand in 2019 report smoking less than daily.

## 4. Discussion

We expanded on a previous study [3] to further examine market share for popular cigarette brands in the United States from 2014 to 2019; an important addition is the analysis of menthol subbrands, the only allowable cigarette flavoring post-TCA and a source of demonstrated public health inequities. The 10 years after the passage of the TCA have been a time of tremendous change in the tobacco product landscape, with mergers, acquisitions, and product divestments, as tobacco companies sought to diversify their product offerings and expand their non-combustible product options; nevertheless, cigarette remains an important pillar of the industry [13] and the most commonly used tobacco product among US adults [14].

Much of the cigarette market’s upheaval from 2014 to 2019 was due specifically to the consolidation of the marketplace after the landmark merger between Reynolds American and Lorillard. A condition of the companies’ merger by the Federal Trade Commission included a joint divestment of a portfolio of four products to Imperial Brands; these products included Winston, Maverick, and menthol brands Salem and Kool. This transaction ultimately resulted in the Reynolds acquisition of the leading brand Newport [13,15,16]. The parent company’s focus on Newport, historically a strong menthol brand, may have slowed the momentum that Pall Mall and Camel (owned by the same parent company) had experienced over the previous years.

We identified three important shifts in the cigarette market between 2014 and 2019. First, while menthol saw a modest 2.1% increase in the market overall, brands that gained the most market share within the menthol market during this time period were brands that are known for having both menthol and non-menthol styles in their product lineups. While Marlboro declined overall, its market share within the category of menthol cigarettes grew by 18.1%. When compared with Newport, a brand known historically for its menthol styles, Marlboro’s menthol market share grew three times more than Newport’s and by 2019, holding only 5% less share of the menthol market than Newport. In the modern cigarette marketplace, the availability of menthol in a tobacco brand’s product lineup is important to grow a brand’s market share [8], as declines in cigarette consumption overall are due to the declines in the use of non-menthol cigarettes, while menthol cigarettes gain market share [10].

This pattern contributed to the second shift we observed, also occurring among discount cigarette brands such as Basic and L&M. Discount brands, in general, have been thriving in the marketplace, despite aggressive price promotion among premium brands after the 2009 FET increase on cigarettes [17], but we observed that menthol styles, in particular, are greatly contributing to increases in market share for many of these brands. Basic’s menthol styles grew twice as much as the brand overall (167% vs. 81.0%); although the brand’s overall market share has not returned to the heights of 6.1% in 2002, both menthol and non-menthol styles show a marked improvement from the previous 12 years [3]. L&M, which did not make the top 10 brands between 2002 and 2013, also experienced almost twice as much growth in their menthol styles than their non-menthol (49.1% vs. 27.1%). The affordability of these brands, combined with the availability of menthol in their lineups, may be appealing to the price-conscious consumer for whom premium branding is not a priority. Research continually demonstrates that while smoking has declined in the United States as a whole, there are certain populations, in particular, the economically disadvantaged, on whom the burden of tobacco use rests [18,19]. Relatedly, our study found that daily smoking was more common among users of low and moderately priced brands, including L&M and Basic. Daily smokers, who are likely more nicotine dependent than nondaily smokers, may look for alternative, less expensive brands, as cigarette prices have increased over time. This may explain the shift toward these more affordable brands among daily smokers [20].

Finally, we observed that the two premium brands, Natural American Spirit and Winston, which used “natural” descriptors in their advertising or on their packaging during this time period, also experienced increased market share. Abundant research exists today regarding the persistence of consumer perceptions about reduced harm from such natural descriptors. However, for more than half of the study period, from 2014 through September 2017, both brands were still permitted to use natural descriptors in advertising and on their packaging, as neither company had yet entered into agreements with the FDA. Winston had used “additive-free” as part of its infamous “no-bull” advertising campaign for at least a decade prior to its 2015 acquisition by ITG. The declining brand subsequently underwent a substantial rebranding effort that included an image refresh, which included “naturally smooth” on its packaging, and a variety of promotions including coupons and sweepstakes. Initially, Winston discontinued the practice of using natural descriptors in its advertising after the 2017 FDA agreement, although “naturally smooth” remained on the pack. Natural American Spirit was permitted by FDA to keep “Natural” in its brand name and to use a variety of other phrases alluding to the product’s naturalness, environmental consciousness, and purity, including “Tobacco and Water” [21,22,23,24]. Natural American Spirit grew consistently in market share since 2002 [3] and managed to maintain its position as a “real” product with “simple” ingredients, likely playing a part in its 97.0% overall market share growth between 2014 and 2019. The brand’s menthol styles too experienced a similar increase of 88.3%.

Through its agreements with Natural American Spirit and Winston, the FDA has inadvertently allowed brands to continue to build on a legacy of years of “natural” descriptors and to skirt FDA regulations through the use of words that have a known halo of reduced harm. Cigarette brands exploit expanding awareness of and concern with environmental health, appealing to consumers in the same way that “organic” labels might affect their purchasing decisions for other products. Similarly, in today’s product marketplace, cigarettes claiming to be made of only “tobacco and water” and “100% plant-based menthol” [16] may provide for some a positive contrast to newer products, such as e-cigarettes and nicotine pouches, that claim synthetic, non-tobacco-derived nicotine. It is well known that consumers of tobacco products continually underestimate the harms due to combustion and overestimate the harms of additives [25,26,27], a fact taken advantage of by Natural American Spirit, Winston, and other cigarette brands in the “natural” category such as Nat’s and Manitou [28], all of which currently use “tobacco and water” in their advertising [22].

Menthol has played an outsized role in the growth of many cigarette brands’ market share increases from 2014 to 2019, providing further evidence that menthol is slowing the decline of the cigarette market. The evidence against exempting menthol, the only flavor exempted from FDA’s cigarette flavor ban, is substantial, and the calls to ban it are unabating. This analysis gives us further reason to continue these calls. However, with some optimism, it must be noted that more than 10 years after Congress exempted menthol in the TCA’s flavor ban in cigarettes [29,30,31], a bright spot may be on the horizon concerning menthol. In April 2021, the FDA asserted its commitment to institute a menthol ban on cigarettes by the end of April 2022 [31]. While it has taken more than a decade of deliberation for the agency to reach its conclusion on menthol, the FDA indicated its decision was based on the accumulation of scientific evidence during this time. Further, the agency has stated that its decision to ban menthol will build on the evidence that supported prior decisions to ban other flavors in 2009 [31]. While there have been continuing calls for a menthol ban for over a decade, the FDA has indicated that the weight of scientific evidence collected since 2009 now offers sufficient supporting evidence to ban menthol for the protection of public health. While tobacco industry litigation is likely, it is believed that a menthol ban would be upheld given this accumulation of evidence [30].

It should be noted that the US, a non-party to the WHO Framework Convention on Tobacco Control, is somewhat unique in its need for continued surveillance of brand preference and market share. Spain, on the other hand, is an example of a country that is a party to the WHO Framework Convention. An analysis of Spain’s tobacco regulation indicates that the country’s stringency has, over time, made tobacco brands’ differentiation through marketing impossible and has resulted in the “homogenizing” of the diffusion process, negating tobacco brands’ ability to differentiate or innovate based on marketing alone [32]. Compared with many countries that adhere to the WHO Framework, the US has comparatively lenient regulation of tobacco products, and US tobacco control researchers and advocates continually call for more regulation to restrict the product attributes on which brands compete, such as flavors, color, pack size, price, and a variety of descriptors, in an effort to have an impact on smokers in the US [33]. The contrast between a country such as Spain that has stronger tobacco regulation than that of the US illustrates clearly that when governments impose a near-total prohibition of advertising and promotion of tobacco, as well as require large pictorial and text warnings on cigarette and smokeless tobacco products, enforce a prohibition on misleading packaging and labeling, and bans on characterizing flavors and a variety of problematic ingredients [34], there can be almost no differentiation between the products. Thus, these shifts in cigarette brand preference we see in the US and report on here may disappear one day with increased regulation of the unique attributes of tobacco products.

This study has several limitations stemming from the nature of the data. Using a single, dichotomous survey item to assess whether the cigarettes respondents smoked during the past 30 days were menthol flavored could induce measurement error if those cigarettes were not their usual brand and/or respondents smoked a mixture of menthol and non-menthol cigarettes (in the latter case, some may have been unsure which option to select). Moreover, as noted above, the NSDUH data only allow for analyses of brand families, rather than subbrands, as they do not contain information on precise brand variants. As this study is based on cross-sectional data, an additional limitation is that we were unable to measure the extent to which smokers may be switching between brands over time.

Some cigarette smokers reported a brand, 305s, that manufactures both cigarettes and little cigars. We could not tell from the NSDUH data which product respondents were using. However, that cigarette smokers might inadvertently report a product that is technically a little cigar is unsurprising, since filtered cigars differ very little physically from cigarettes [35], and are common cigarette substitutes.

Additionally, past research has shown associations between consumer characteristics and market trends [36,37], which we did not assess in this study. Future research should attempt to determine the demographic correlates of current market trends and assess whether those correlates have changed over time. Additionally, we did not distinguish between single product tobacco users (i.e., cigarettes only), and poly-tobacco users, as the purpose of this analysis did not require such distinction. This may be an area for future research, as there may be brand preferences and patterns distinct to each group that are not evident here and may be important for tobacco regulation and intervention.

Finally, the time period from 2014 to 2019 is before the arrival of the COVID-19 pandemic. How lockdowns, stress and isolation, unemployment, government stimulus payments, supply chain issues, and other concerns related either directly or tangentially to COVID-19 have or will affect the cigarette marketplace are yet to be realized. There are many opportunities for future research as we start to observe the first clues of the COVID-19 cigarette marketplace; for example, the 2020 FTC Cigarette Report notes the first increase in cigarette sales in 20 years [38]. In addition, while the economic downturn of 2020 in the US was relatively short, compared with that of 2008–2009, economic disparities in the US are vast, and the financial impacts on individuals have been varied. It will be worthwhile to explore in future research whether a notable pattern of cigarette brand switching begins in 2020 and continues, perhaps as a means to lessen economic pressures resulting from the downturn.

## 5. Conclusions

The importance of monitoring the shifts in cigarette brand preferences cannot be overstated, as the identification and evaluation of these trends are key to understanding the cigarette marketplace. Many factors can shift cigarette brand preference, and surveillance is an important tactic to inform regulatory strategy. Future research should continue to focus on the trends that influence the market share of cigarette brands, as the next 5–10 years will likely continue to look very different than the current study.

## Figures and Tables

**Table 1:** Market share of all brands, regular or menthol, appearing in NSDUH’s Top 15 in the years 2014 to 2019.

Brand	2014	2015	2016	2017	2018	2019	% Change 2014–2019
Marlboro	39.59	39.79	37	39.09	39.54	38.41	−3.0%
Newport	10.41	11.17	11.94	12.58	12.57	12.24	17.6%
Pall Mall	8.23	8.4	7.95	7.64	8.73	7.98	−3.0%
Camel	8.19	7.89	7.39	7.69	7.99	7.09	−13.4%
American Spirit	1.69	2.25	2.59	2.83	2.91	3.33	97.0%
L&M	2.55	2.59	2.9	2.5	2.97	3.24	27.1%
Winston	1.97	1.98	2.37	1.93	1.77	2.29	16.2%
Eagle	0.14	0.13	0.59	1.14	0.76	2.11	1407.1%
Basic	1.16	1.18	1.33	1.33	1.15	2.10	81.0%
KOOL	2.22	1.32	2.04	1.63	1.88	1.92	−13.5%
Top (roll-your-own)	1.56	1.42	1.52	1.54	1.27	1.26	−19.2%
Pyramid	1.32	1.42	1.09	0.67	0.78	1.21	−8.3%
USA Gold	1.31	2.04	1.88	1.42	0.97	0.92	−29.8%
305s	1.13	1.43	1.06	1.1	1.37	0.84	−25.7%
Virginia Slims	1.09	1.4	1.52	0.78	0.78	0.74	−32.1%
All Other Brands	17.44	15.59	16.83	16.13	14.56	14.32	

**Table 2:** Market share of brands’ menthol styles appearing in NSDUH’s Top 10 in the years 2014 to 2019.

Brand	2014	2015	2016	2017	2018	2019	% Change 2014–2019
Newport	28.73	31.89	32.40	31.16	31.80	30.57	6.4%
Marlboro	21.80	23.12	20.69	23.14	23.28	25.75	18.1%
Camel	8.36	8.15	8.81	8.36	9.39	7.77	−7.1%
Pall Mall	7.97	6.45	5.31	5.43	7.01	8.31	4.3%
KOOL	6.76	4.26	6.43	4.77	5.80	5.52	−18.3%
Salem	3.90	2.90	2.10	1.77	1.77	1.55	−60.3%
Virginia Slims	2.40	3.22	4.00	1.33	1.27	1.00	−58.3%
L&M	2.22	1.91	1.76	3.64	3.19	3.31	49.1%
Misty	1.47	1.98	0.96	1.00	0.45	0.88	−40.1%
Maverick	1.45	2.04	1.45	1.91	2.02	1.19	−17.9%
USA Gold	0.99	1.96	1.72	1.07	0.61	0.61	−38.4%
American Spirit	0.60	0.84	1.22	1.26	1.12	1.13	88.3%
Basic	0.74	0.45	1.09	1.13	0.20	1.98	167.6%
Top (Roll-Your-Own)	0.70	1.34	1.37	1.26	0.97	1.18	68.6%
All Other Brands	11.91	9.49	10.69	12.77	11.12	9.25	

**Table 3:** Demographic profile of smokers of the top 10 brands, 2014 and 2019.

	Marlboro		Newport		Pall Mall		Camel		American Spirit		L&M		Winston		Eagle		Basic		KOOL	
	2014	2019	2014	2019	2014	2019	2014	2019	2014	2019	2014	2019	2014	2019	2014	2019	2014	2019	2014	2019
**Age**																				
12 to 17	2.75	1.38	3.02	1.22	1.15	0.55	2.62	2.05	2.26	1.11	1.42	0.59	0.36	0.73	0.00	0.29	2.45	2.25	1.57	0.38
18 to 25	20.34	15.06	24.16	14.76	6.35	3.80	29.58	19.41	26.62	18.92	20.61	11.95	1.05	2.70	9.50	4.40	5.53	3.11	11.26	5.44
26 to 49	51.94	52.06	55.42	60.87	37.73	33.34	52.83	66.15	50.94	47.34	47.52	61.22	18.59	16.00	18.75	29.44	26.39	15.88	28.71	41.40
50 +	24.95	31.50	17.40	23.14	54.77	62.33	14.96	12.40	20.17	32.63	30.45	26.24	80.00	80.56	71.75	65.87	65.62	78.76	58.46	52.78
**Gender**																				
Male	56.71	54.78	50.97	51.73	58.74	52.75	60.00	60.71	69.97	61.39	55.22	54.33	58.45	58.65	22.30	51.31	47.97	59.65	51.89	53.43
Female	43.29	45.22	49.04	48.27	41.26	47.25	40.00	39.29	30.03	38.61	44.78	45.67	41.55	41.35	77.70	48.69	52.03	40.35	48.11	46.57
Ethnicity																				
White NH	74.86	74.63	27.35	30.98	83.57	84.07	75.99	64.56	77.48	81.39	87.99	80.77	89.53	90.09	95.03	86.01	76.22	81.56	41.82	30.84
Black NH	2.85	2.52	52.32	50.62	7.31	5.97	3.66	3.24	5.48	2.16	3.80	7.58	4.69	3.65	0.0	4.77	9.62	2.54	45.37	54.05
Hispanic	15.65	14.79	15.67	14.01	5.56	3.79	15.45	22.92	11.08	7.59	2.88	9.20	1.86	3.01	3.76	2.96	9.87	12.80	8.25	6.45
Other	6.65	8.06	4.66	4.39	3.57	6.17	4.89	9.28	5.96	8.86	5.33	2.46	3.92	3.25	1.21	6.25	4.29	3.10	4.57	8.67
**Income**																				
29,999 or less	32.59	28.50	54.39	49.67	46.19	35.29	36.09	28.86	28.50	32.89	56.47	34.68	37.14	26.53	72.32	50.92	63.21	70.83	46.94	47.03
30,000–74,999	40.40	38.68	32.92	33.98	36.93	40.92	41.53	41.66	38.67	32.28	31.68	45.85	45.67	34.91	25.39	39.46	23.10	23.44	34.15	41.57
75,000 +	27.01	32.82	12.69	16.35	16.87	23.79	22.38	29.48	32.82	34.83	11.85	19.46	17.19	38.56	2.28	9.61	13.68	5.73	18.91	11.39
**Daily/Nondaily Smk**																				
Nondaily Smk	42.38	41.09	47.93	46.96	29.00	26.45	48.35	48.55	61.66	59.11	24.20	25.50	34.79	32.88	35.04	21.47	44.37	32.84	33.25	45.35
Daily Smk	57.62	58.91	52.07	53.04	71.00	73.55	51.65	51.45	38.34	40.89	75.80	74.50	65.21	67.12	64.96	78.53	55.63	67.16	66.75	54.65

Abbreviations: NH, Non-Hispanic; Smk, smoking.

## Data Availability

This study was a secondary analysis of data collected for the 2014–2019 NSDUH. Data and details regarding the NSDUH methodology are available at the Substance Abuse and Mental Health Data Archive (https://www.datafiles.samhsa.gov/ (25 January 2022).
